# Comparative Cytotoxicity Evaluation of Heat-Assisted *vs* Cold Water Extractions of Six Medicinal Fungi against Breast and Lung Cancer Cells

**DOI:** 10.17113/ftb.62.02.24.8424

**Published:** 2024-06

**Authors:** Min-Jia Ng, Neng-Yao Goh, Chon-Seng Tan, Muhammad Fazril Mohamad Razif, Hui-Yeng Yeannie Yap, Boon-Hong Kong, Shin-Yee Fung

**Affiliations:** 1 Medicinal Mushroom Research Group (MMRG), Department of Molecular Medicine, Faculty of Medicine, Universiti Malaya, 50603 Kuala Lumpur, Malaysia; 2 LiGNO Biotech Sdn. Bhd., Jalan Perindustrian Balakong Jaya 2/2, Taman Perindustrian Balakong Jaya 2, 43300 Balakong Jaya, Selangor, Malaysia; 3 Division of Applied Biomedical Sciences and Biotechnology, School of Health Sciences, IMU University, Jalan Jalil Perkasa, Bukit Jalil, 57000 Kuala Lumpur, Malaysia; 4 Center for Natural Products Research and Drug Discovery (CENAR), Level 3, Research Management and Innovation Complex Universiti Malaya, Universiti Malaya, 50603 Kuala Lumpur, Malaysia; 5 Universiti Malaya Centre for Proteomics Research (UMCPR), Universiti Malaya, 50603 Kuala Lumpur, Malaysia; § These authors contributed equally to this work

**Keywords:** fungal extract, heat-assisted water extraction, cold water extraction, protein, carbohydrate and phenolic compositions, cytotoxicity

## Abstract

**Research background:**

Preparation of medicinal fungi for experimental purposes usually involves the extraction and determination of the quality and quantity of bioactive compounds prior to the biological experiment. Water, a common polar solvent, is usually used for traditional preparations for consumption. The application of high temperatures during water extraction can affect the chemical composition and functional properties of the extracts. Therefore, the aim of this study is to compare the differences in composition between extracts obtained with heat-assisted and cold water extractions of six selected species of fungi (*Lignosus rhinocerus*, *Ophiocordyceps sinensis*, *Inonotus obliquus*, *Antrodia camphorata*, *Phellinus linteus* and *Monascus purpureus*) and their cytotoxicity against human lung and breast cancer cells.

**Experimental approach:**

The extracts obtained with heat-assisted and cold water extraction of six species of fungi were analysed to determine their protein, carbohydrate and phenolic contents. Their cytotoxicity was tested against lung (A549) and breast (MCF-7 and MDA-MB-231) cancer cell lines. The most potent extract was further separated into its protein and non-protein fractions to determine their respective cytotoxicity.

**Results and conclusions:**

The cytotoxicity of the different extracts obtained with heat-assisted and cold water extraction varied. Comparing the two extractions, the cold water extraction resulted in a significantly higher yield of proteins (except *M. purpureus)* and phenolic compounds (except *A. camphorata*), while the extracts of *I. obliquus* and *M. purpureus* obtained with heat-assisted extraction had a significantly higher carbohydrate mass fraction. Notably, the cold water extract of *I. obliquus* showed cytotoxicity (IC_50_=(701±35) µg/mL), which was one of the highest of the extracts tested against A549 cells. The cold water extract of *I. obliquus* was selected for further studies. Our results showed that cold water extracts generally have higher cytotoxicity against selected human cancer cell lines, with the exception of *O. sinensis* and *A. camphorata* extracts.

**Novelty and scientific contribution:**

This study reports the advantage of cold water extracts of fungi over those obtained with heat-assisted extraction in terms of cytotoxicity against human cancer cell lines and emphasises the role of extraction conditions, particularly heat, in influencing chemical composition and cytotoxic effects.

## INTRODUCTION

Medicinal fungi have a long history of use in traditional and complementary medicine for their therapeutic properties such as antimicrobial, antioxidant, anti-inflammatory and anticancer (*1*). These are attributed to the presence of various bioactive compounds such as polysaccharides, lectins, lanostanoids, alkaloids and phenolics (*1*). Many fungi have attracted considerable attention due to their rich and diverse chemical composition.

In this study, we have focused on six remarkable species of medicinal fungi mainly found in Asia: *Lignosus rhinocerus*, *Ophiocordyceps sinensis*, *Inonotus obliquus*, *Antrodia camphorata*, *Phellinus linteus* and *Monascus purpureus*. *Lignosus rhinocerus*, also known as the Tiger Milk mushroom, is a rare and highly sought-after medicinal fungus native to Southeast Asia. It has been used by indigenous tribes to treat respiratory conditions, arthritis and cancer (*2*, *3*). The Chinese caterpillar fungus, scientifically known as *O. sinensis*, has several bioactivities that have earned it immense recognition in traditional Chinese medicine. Its discovery was initially prompted by its parasitic relationship with moth caterpillars (*4*). *O. sinensis* is often used to treat asthma, bronchial inflammation and lung ailments, and is traditionally considered to support longevity. The distribution of *O. sinensis* is mainly limited to the Tibetan Plateau and neighbouring areas.

Chaga, scientifically known as *I. obliquus*, grows in the cold birch forests of Siberia (*5*). Its antioxidant and anti-inflammatory effects are enhanced by bioactive components such as polysaccharides and betulinic acid (*6*). *Antrodia camphorata* from Taiwan was found on the decomposing wood of *Cinnamomum kanehirai* (*7*). It is known for its hepatoprotective and potentially anticancer properties (*7*). *Phellinus linteus*, also known as Sang Hwang, is a fungus that is widely distributed in Asia, especially in Korea and China (*8*). It is traditionally used to regulate blood glucose levels, improve blood circulation, protect the liver and strengthen the immune system (*8*). *Monascus purpureus*, or red yeast, is found in red yeast rice as well as in fermented foods and beverages made with red yeast rice, especially in China, Taiwan and Korea (*9*). It is known to produce vibrant red pigments that are commonly used as food colorants and has cholesterol-lowering properties due to the compound monacolin K (*10*).

Natural compounds from living organisms such as plants, fungi and microbes form the basis for the health-promoting properties of many natural remedies that support overall well-being (*11*). The preparation of these natural remedies and medicinal preparations dates back to ancient times. Water-based preparation methods, including decoction and hot infusion, are the two most commonly used approaches. In a decoction, the harder parts of the raw ingredients are boiled in water, while in a hot infusion, the softer parts are gently steeped in hot water. These methods aim to extract beneficial compounds for therapeutic purposes. While the practice of preparing decoctions with hot water has been extensively studied, the potential effects of hot (steeped in hot water) and cold (soaked in cold water for 12 to 24 h) water extractions and their differences in composition and functions have not been thoroughly investigated, especially for the selected fungi used in this study. Therefore, two types of water extraction methods were used in our study: heat-assisted water extraction and cold water extraction, which serve as models for hot and cold water infusions.

Different extraction temperatures have the potential to yield different functional compounds with different bioactivities (*12*). This study aims to evaluate and compare the differences in the composition (carbohydrates, proteins and phenolic compounds) of the extracts of six selected fungi obtained with cold water and heat-assisted extractions, and to evaluate the cytotoxic effects of these extracts *in vitro* on different lung and breast cancer cell lines. Of the selected medicinal fungi, those that exhibit higher cytotoxicity were further separated to identify the specific fraction containing potentially cytotoxic compounds of interest that can be utilised for future drug development or prophylactic treatments.

## MATERIALS AND METHODS

### Fungal samples

*Lignosus rhinocerus* TM02^®^ (batch no: PL/1107/020), *Ophiocordyceps sinensis* OCS02^®^ (batch no: OC/21/001-1122), *Inonotus obliquus* (batch no: CG/20/003-1122), *Antrodia camphorata* (batch no: AC/21/001-1122), *Phellinus linteus* (batch no: SH/21/002-1122) and *Monascus purpureus* (batch number not applicable, single cultivar) were obtained from LiGNO Biotech Sdn Bhd (Selangor, Malaysia). These fungi were cultivated using their proprietary solid stage fermentation technology with rice-based medium as substrate. PCR amplification coupled with sequence analysis of their respective internal transcribed spacer (ITS) region was used to verify their identity (*13*, *14*).

### Cell lines and cell culture

Human lung carcinoma epithelial cells A549 (CRM-CCL-185™, ATCC, Manassas, VA, USA) and human breast adenocarcinoma MCF-7 (HTB-22™, ATCC) were cultured in HyClone RPMI-1640 (Cytiva, Marlborough, MA, USA) supplemented with *φ*=10 % foetal bovine serum (FBS) (Tico Europe Ltd., Amstelveen, The Netherlands). Human breast adenocarcinoma MDA-MB-231 (CRM-HTB-26™, ATCC) was cultured in DMEM (Nacalai Tesque Inc., Kyoto, Japan) supplemented with *φ*=10 % FBS. Nontumorigenic human lung epithelial cell line NL20 (CRL-2503, ATCC) was cultured in Ham’s F-12 medium (Nacalai Tesque Inc.) supplemented with *φ*=10 % FBS. All cell lines were incubated at 37 °C in a 5 % CO_2_ incubator (Heracell 150; Marshall Scientific, Hampton, VA, USA). Cells were subcultured every 3 to 4 days with 0.25 % (*m*/*V*) trypsin-EDTA solution (Biosera, Cholet, France).

### Extraction methods

Fungal extracts were prepared using two different water extraction methods: cold water and heat-assisted water extractions.

#### Cold water extraction

Dried crude fungal powder 30 g was dissolved in a 600 mL of distilled water. The solution was stirred for 24 h at 4 °C in a cold room. The mixture was then centrifuged at 8000×*g* for 30 min at 4 °C in a Sorvall Biofuge PRIMO R refrigerated centrifuge (Thermo Fisher Scientific Inc., Waltham, MA, USA) to remove any insoluble compounds. The resulting supernatant was filtered through Whatman filter paper (grade 1, pore size=1.1·10^−5^ m; GE Healthcare, Chicago, IL, USA) and subsequently freeze-dried. The cold water extract was stored at −20 °C.

#### Heat-assisted water extraction

A total of 100 g crude fungal powder was dissolved in 500 mL of warm water (50–60 °C). After all crude fungal powder was thoroughly hydrated, the volume was made up to a final volume of 1 L with boiled distilled water. The final temperature of the solution was 80 °C. The mixture was shaken periodically at room temperature for 3 h. The mixture was transferred to a centrifuge tube and centrifuged (Sorvall Biofuge PRIMO R refrigerated centrifuge; Thermo Fisher Scientific Inc.) at 5000×*g* for 30 min. The supernatant after heat-assisted water extraction was then collected, freeze-dried and stored at -20 °C.

Frozen solutions of both *L. rhinocerus* and *O. sinensis* were removed and thawed after 12 h at room temperature (25 °C). Precipitates were observed after thawing, and the mixture was centrifuged at 5000×*g* for 30 min. The supernatant was then transferred to a new tube. The remaining precipitate was washed with distilled water and spun at 5000×*g* for 30 min. The water used for rinsing was removed and the rinsed precipitate (the obtained precipitate was only soluble up to 80 °C) was resuspended in 200 mL of distilled water. The precipitate was freeze-dried and stored at -20 °C until further use.

### Quantification of protein, carbohydrate and phenolic content of extracts

The 2-D Quant kit (GE Healthcare) was used to quantify the protein content in the samples following the manufacturer's instructions.

The phenol-sulfuric acid method was used to quantify the total carbohydrate content (*15*). Briefly, 200 μL of 5 % phenol (Merck KGaA, Darmstadt, Germany) were added to 200 μL of the sample, followed by the addition of 1 mL of 95 % sulfuric acid (Friendemann Schmidt Chemical, Kuala Lumpur, Malaysia). After thorough mixing, the mixture was incubated at room temperature for 20–30 min. The absorbance of the mixture was measured at 490 nm using a UV–Vis spectrophotometer (SpectraMax^®^ ABS Plus, Molecular Devices, San Jose, CA, USA).

The Folin-Ciocalteu assay was used to quantify the phenolic content (*16*). A volume of 10 μL of the extract was mixed with 500 μL of Folin-Ciocalteu phenol reagent (Merck KGaA) and incubated for 5 min at room temperature. Then, 350 μL of sodium carbonate (Sigma-Aldrich^®^, Merck KGaA) (115 μg/mL) were added to the mixture and incubated for 2 h. Gallic acid (Sigma-Aldrich^®^, Merck KGaA) was used as a standard at concentrations ranging from 20 to 200 μg/mL and the absorbance was measured at 765 nm using a UV–Vis spectrophotometer (SpectraMax^®^ ABS Plus, Molecular Devices).

### Ammonium sulfate precipitation of the cold water extract of I. obliquus

Ammonium sulfate (Friendemann Schmidt Chemical) at 100 % saturation was used to precipitate the protein and non-protein compounds of the cold water extract of *I. obliquus* with stirring for 1 h at 4 °C. Centrifugation was used to separate the precipitated protein pellet from the supernatant (containing non-protein compounds). The protein and non-protein compounds were then desalted using the Vivaspin^®^ centrifugal concentrator 15R (MWCO 5 kDa) (Sartorius AG, Göttingen, Germany).

### Cytotoxicity assay

The MTT (2,5-diphenyl-2H-tetrazolium bromide) assay (Merck KGaA) was used to examine the cytotoxicity of extracts against a panel of human cancer cell lines, including MCF-7 (breast cancer, ER^+^ and PR^+^), MDA-MB-231 (breast cancer, ER/PR^-^ and HER2^-^) and A549 (lung cancer). The cytotoxicity of both protein and non-protein components of the cold water extract of *I. obliquus* were tested against lung carcinoma A549 and its respective non-tumorigenic cell line, NL20. On day 1, each cell line was seeded into a 96-well plate. The cells were treated with both extracts after 24 h and incubated for 72 h in a CO_2_ incubator (Heracell 150; Marshall Scientific). Then, 20 µL of 5 mg/mL MTT reagent (Sigma-Aldrich^®^, Merck KGaA) were added into each well and incubated for 3 h at 37 °C. After incubation, the MTT reagent was removed and 200 µL of DMSO (Friendemann Schmidt Chemical) were added to each well. The 96-well plate was shaken for 15 min on an orbital shaker (Stuart SSM3 Mini Gyratory Rocker; Bibby Sterilin Ltd, Stone, UK). The absorbance was then measured at 570 nm for 1 h. IC_50_ was obtained through plotted survival curve of cells in each treatment. Selectivity index is equal to the IC_50_ of non-tumorigenic cell lines divided by the IC_50_ of cancer cell lines (*17*).

### Statistical analysis

The Shapiro-Wilk test was used to assess the data normality, and the Levene’s test was used to determine the equality of variances. Statistical significance between the protein, carbohydrate and phenolic content of the cold water extracts and the supernatant and precipitate of the extracts of *L. rhinocerus* and *O. sinensis* obtained with heat-assisted extraction was evaluated using analysis of variance (ANOVA) with Tukey’s HSD *post-hoc* test. Since the data set of protein content of extracts of *M. purpureus* obtained with cold water and heat-assisted extraction did not meet the normality assumption, the two groups were compared using the non-parametric Mann-Whitney *U*-test. The protein, carbohydrate and phenolic content of the remaining groups was determined between the two types of extractions using the parametric Student’s *t*-test. All data are expressed as mean value±standard deviation (S.D.). A *p*-value of less than 0.05 was considered statistically significant. The data were analysed using Microsoft^®^ Excel (*18*), IBM^®^ SPSS^®^ Statistics software v. 26.0 (*19*) and GraphPad Prism^®^ v. 6.01 (*20*).

## RESULTS AND DISCUSSION

### Protein, carbohydrate and phenolic content of fungal extracts

Our analysis showed significant differences between the two types of extracts in protein and phenolic content (Table 1). Most cold water extracts had a significantly higher protein mass fraction than those obtained with heat-assisted extraction. An exception was observed in *M. purpureus* extracts, where both types of extracts had similarly low protein mass fraction, probably due to an inherently low protein mass fraction or measurements falling below the detection limit. As most proteins are sensitive to high temperatures, cold water extraction has been shown to be favourable for maintaining protein integrity (*21*). It is crucial to note that the heat-assisted water extraction used in this study does not involve boiling, unlike previously reported hot water extraction (*22*). Nonetheless, both methods involve high temperatures that can potentially damage thermolabile compounds.

**Table 1:** Protein, carbohydrate and phenolic mass fractions of various fungal extracts

Fungus	Extraction	*w*(protein)/(mg/g)	*w*(carbohydrate)/(mg/g)	Total phenolics as *w*(GAE)/(mg/g)
*Lignosus rhinocerus*	CWE	(4.1±0.8)^a^	(708±42)^a^	(1.08±0.04)^a^
	HAWE_supernatant_	(0.5±0.2)^b^	(856±107)^a^	(0.91±0.05)^b^
	HAWE_precipitate_	(0.5±0.1)^b^	(764±126)^a^	(0.16±0.02)^c^
*Ophiocordyceps*	CWE	(2.3±0.3)^a^	(446±70)^a^	(1.9±0.5)^a^
*sinensis*	HAWE_supernatant_	(0.18±0.02)^b^	(760±165)^a^	(0.46±0.07)^b^
	HAWE_precipitate_	(0.28±0.09)^b^	(739±126)^a^	(0.08±0.01)^b^
*Antrodia camphorata*	CWE	(0.70±0.03)^a^	(626±21)^a^	(10.1±0.2)^a^
	HAWE	(0.24±0.06)^b^	(635±27)^a^	(11.0±0.4)^b^
*Phellinus linteus*	CWE	(4.6±1.1)^a^	(513±18)^a^	(2.04±0.08)^a^
	HAWE	(1.4±0.7)^b^	(742±178)^b^	(0.41±0.01)^b^
*Inonotus obliquus*	CWE	(3.3±0.1)^a^	(431±6)^a^	(4.90±0.09)^a^
	HAWE	(1.5±0.3)^b^	(735±14)^b^	(1.41±0.10)^b^
*Monascus*	CWE	(0.01±0.06)^a^	(516±42)^a^	(10.2±0.2)
*purpureus*	HAWE	(0.18±0.03)^a^	(837±93)^b^	NA

Protein and carbohydrate mass fractions are expressed on dry mass basis. GAE=gallic acid equivalents. Data are expressed as mean value±S.D. (*N*=9). Different letters in superscript denote significant differences (p˂0.05) among the respective fungal extracts. Statistical analysis was conducted using one-way analysis of variance (ANOVA) with Tukey’s HSD *post-hoc* analysis among the respective *L. rhinocerus* and *O. sinensis* extracts. The statistical significance of the remaining species was determined using Student’s *t*-test. NA=not available, CWE=cold water extraction, HAWE=heat-assisted water extraction

The cold water extracts had significantly higher phenolic content than those obtained with heat-assisted extraction, with the exception of *A. camphorata* (Table 1). Phenolic compounds have the ability to undergo redox reactions as hydrogen atom donors or reducing agents, which serve to neutralise electrons of reactive oxygen species (ROS) (*23*). It is worth noting that the heat-assisted extraction of *I. obliquus* and *M. purpureus* resulted in significantly higher mass fractions of carbohydrates in the extracts than cold water extraction. Higher temperature during heat-assisted water extraction probably increases carbohydrate extraction by reducing the solvent viscosity, improving the solubility of carbohydrates and facilitating diffusion in water (*24*).

### Cytotoxicity of fungal extracts towards lung and breast cancer cells

Carbohydrates, proteins and phenolic compounds in fungi, including beta-glucans, serine proteases, and resveratrol, have known cytotoxic bioactivities (*25*-*27*). We hypothesised that different compositions of the extracts, due to different extraction conditions (Table 1), might influence the cytotoxicity of selected fungal extracts.

**Fig. 1:**
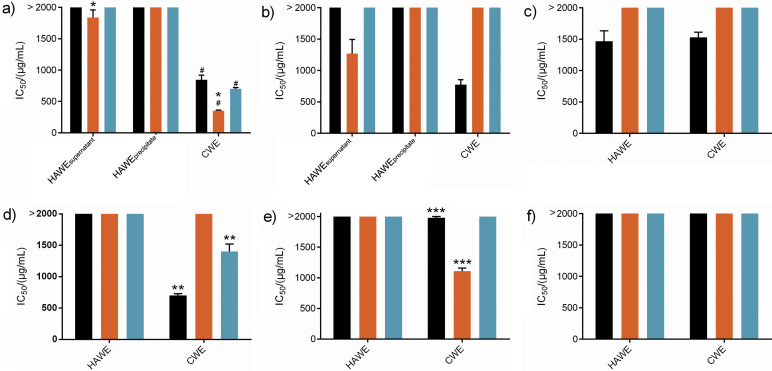
Graphical representations of the IC_50_ values for each fungal extract: a) *L. rhinocerus*, b) *O. sinensis*, c) *A. camphorata*, d) *I. obliquus*, e) *P. linteus* and f) *M. purpureus*, with respect to the tested cancer cell lines: A549 lung carcinoma (black), MCF-7 breast carcinoma (orange), and MDA-MB-231 breast carcinoma (blue). Data are expressed as mean value±S.D. (*N*=9). All IC_50_ values exceeding 2000 µg/mL are represented as >2000 µg/mL in the graphs, with no error bars shown. # indicates significant differences (p˂0.05) of *L. rhinocerus* CWE IC_50_ values between the tested cell lines based on one-way ANOVA followed by Tukey’s HSD *post-hoc* test. The remaining statistical analysis was determined using Student’s *t*-test. * denotes significant differences (p˂0.05) of IC_50_ values between *L. rhinocerus* CWE and HAWE against MCF-7 cell line. ** and *** indicate significant differences (p˂0.05) of IC_50_ of *I. obliquus* and *P. linteus,* respectively, between the cell lines with IC_50_ <2000 µg/mL. CWE=cold water extraction, HAWE=heat-assisted water extraction

Fig. 1 illustrates the half-maximal inhibitory concentration (IC_50_) of the extracts against A549, MCF-7 and MDA-MB-231 cell lines. Our results for *L. rhinocerus* are consistent with previous studies (*12*, *28*), in which the cold water extract of this fungus showed higher cytotoxicity than its hot water extract. This suggests that the cytotoxicity of *L. rhinocerus* may be due to the thermolabile water-soluble compounds that are altered or degraded during heat-assisted extraction. Cold water extraction appears to be the most effective method for extracting bioactive (anticancer and anti-inflammatory) compounds from *L. rhinocerus*, as shown by the results of numerous studies (*12*, *29*).

The water extracts of *O. sinensis* had different cytotoxicities against different human cancer cell lines. The cold water extract of *O. sinensis* was more cytotoxic against A549 cells than the extract of *O. sinensis* obtained with heat-assisted extraction, while the supernatant of *O. sinensis* extract obtained using heat had stronger cytotoxicity against MCF-7 cells (Fig. 1). The cold water extract of *O. sinensis* had higher mass fractions of proteins and phenolic compounds than both the supernatant and the precipitate of *O. sinensis* extracts obtained using heat-assisted extraction of (Table 1), which is consistent with previous studies (*30*). This finding suggests that thermolabile compounds, including the proteins and phenolic compounds in the cold water extract of *O. sinensis*, may be the main contributors to the observed cytotoxicity against A549 cells. Considering that both the supernatant and precipitate of the heat-assisted extract of *O. sinensis* have a similar high carbohydrate mass fraction and low mass fractions of proteins and phenolics, the cytotoxic compounds against MCF-7 cells are likely to be carbohydrates and possibly other water-soluble thermostable compounds. Cordycepin, a nucleoside with anticancer properties, could be a candidate as it can be extracted at higher temperatures (up to 70 °C) (*31*). Our results suggest that the cytotoxicity of the *O. sinensis* extracts may depend on the specific cancer cell type and extraction temperature. The differences in the cytotoxic activities of the *O. sinensis* extracts are likely due to the presence of specific compounds in each extract that exhibit selective toxicity towards specific cancer cell lines, possibly targeting different signalling pathways or factors. These differences are due to the physiological properties and signalling mechanisms of cancer cells from different organs.

Both extract types (obtained with cold water or using heat) of *A. camphorata* showed similar cytotoxicity against A549 cell lines, suggesting that their activity is attributed to thermostable water-soluble compounds in the fungi that are retained even after heat-assisted extraction. These cytotoxic components in *A. camphorata* water extracts are likely carbohydrates or phenolic compounds, as both cold water and heat-assisted extraction resulted in similar mass fraction of these metabolites.

Both extract types of *M. purpureus* did not show cytotoxicity (IC_50_>2000 µg/mL) against any of the tested cancer cell lines (Fig. 1). Although the water extracts have a high carbohydrate mass fraction, we hypothesise that the cytotoxic compounds in *M. purpureus* may be non-polar. Previous studies have shown that *M. purpureus* extracts obtained with different solvents, such as the petroleum ether-soluble part of the EtOH extract, contain non-polar monapurones A-C that exhibit strong selective cytotoxic effects on A549 lung cancer cell lines (*32*). The extraction of these cytotoxic compounds can be achieved with solvents of low polarity, such as petroleum ether, suggesting that the bioactive compounds in *M. purpureus* with anticancer properties are either insoluble in water or present in low amounts in the water extract.

The cold water extract of *I. obliquus* showed one of the lowest IC_50_ values against A549 cells, with no significant difference to the cold water extracts of *L. rhinocerus* and *O. sinensis*. Nevertheless, it outperformed the heat-assisted extract of *I. obliquus* and other tested fungal extracts (Fig. 1 and Table S1). Our result is of interest as this is the only reported aqueous extract of *I. obliquus* with a low IC_50_ value. For comparison, results obtained so far were higher (*33*). It is also lower than other extraction methods using organic solvents, such as methanol (IC_50_=(2.0±0.2) mg/mL) (*34*). Water is a solvent with greater polarity than methanol. Therefore, it can extract a larger amount of hydrophilic compounds. We hypothesise that the highly cytotoxic compound for the A549 cell lines is most likely hydrophilic. In another study, the cytotoxicity of an aqueous extract of *I. obliquus* at room temperature against A549 lung cancer cells (IC_50_ was not mentioned) was also reported (*33*). According to our results (Fig. 1d), the bioactive compounds in *I. obliquus* can be effectively extracted under aqueous conditions and are potentially thermolabile. Importantly, none of the extracts from previous studies with *I. obliquus* were obtained by cold water extraction. Therefore, our results prompt further investigation into the specific cytotoxic components responsible for the observed cytotoxicity in the cold water extract of *I. obliquus*.

Overall, with the exception of a few cases mentioned above, our results showed that the cold water extracts had higher cytotoxicity to most tested fungi than the ones obtained using heat (Fig. 1 and Table S1). These results demonstrate the importance of understanding the potency, stability and solubility of specific bioactive compounds to determine the best extraction method for different species of medicinal fungi. The varied cytotoxicity observed between the extracts of different fungi also emphasises the complex nature and potential selectivity of their bioactive compounds towards specific cancer cell lines.

### Cytotoxic activity of water extracts of I. obliquus protein and non-protein components

The cold water extract of *I. obliquus* contained significantly higher mass fractions of proteins and phenolic compounds than the one obtained using heat, suggesting the presence of cytotoxic compounds in the cold water extract. Therefore, we decided to analyse the extract by separating it further into protein and non-protein fractions using ammonium sulphate precipitation. The protein fraction of the cold water extract of *I. obliquus* contained a higher mass fraction of proteins ((76.3±2.3) %) and a lower mass fraction of carbohydrates ((19.0±5.6) %) than the non-protein fraction, which had no detectable protein and a high mass fraction of carbohydrates ((69.2±3.5) %).

**Fig. 2:**
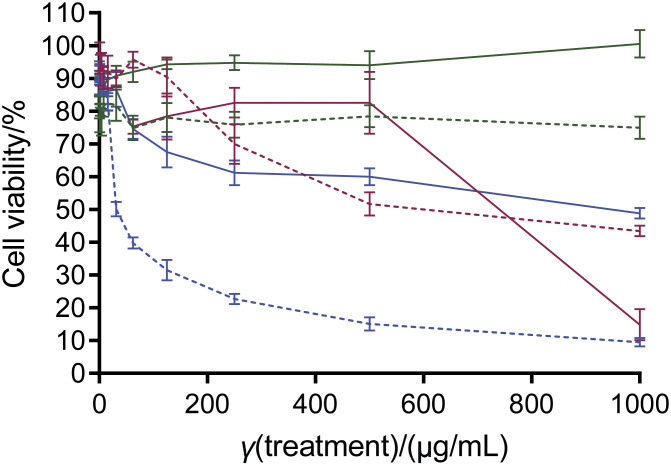
Growth inhibition of A549 lung carcinoma (full line) and NL20 non-tumorigenic (dotted line) cell lines after 72 h of treatment in the MTT assay with the cold water extract of *I. obliquus* (green) and its protein (blue) and non-protein (red) fractions

The analysis showed that the cold water extract of *I. obliquus* has higher cytotoxicity against A549 cells than the protein and non-protein fractions. The non-protein fraction of the cold water extract of *I. obliquus* showed no detectable cytotoxicity (IC_50_>1000 µg/mL). Surprisingly, both the cold water extract of *I. obliquus* and its protein fraction showed significantly higher cytotoxic selectivity towards non-tumorigenic human lung NL20 cells than towards A549 (Table 2 and Fig. 2). Nevertheless, the extract showed significantly lower cytotoxicity to NL20 cells (IC_50_=(549±44) µg/mL) than the protein fraction (IC_50_=(31.4±0.6) µg/mL). In the cold water extract of *I. obliquus*, the non-protein compounds may have counteracted the cytotoxicity exerted by the proteins towards NL20 cells, thus reducing its cytotoxic selectivity towards NL20 (selectivity index 0.8). Overall, this suggests that consuming *I. obliquus* in its entirety could provide a more balanced effect than consuming only the protein fraction. Further research is needed to understand the synergistic interactions between its protein and non-protein fractions by identifying the specific bioactive compounds present in the cold water extract and protein fraction of *I. obliquus* that affect lung non-tumorigenic cells.

**Table 2:** Half maximal inhibitory concentration (IC_50_) of *Inonotus obliquus* cold water extract and its protein and non-protein fractions against A549 lung carcinoma and non-tumorigenic NL20 cells after 72 h of treatment

*Inonotus obliquus*	IC_50_/(µg/mL)		Selectivity
	A549	NL20	index
Cold water extract	(701±35)^a^	(549±44)^c^	0.8
Protein fraction	(931±70)^b^	(31.4±0.6)^d^	0.03
Non-protein fraction	>1000	>1000	-

Data are expressed as mean value±S.D. (*N*=9). Different letters in superscript denote significant difference (p˂0.05). Student's *t*-test was used to compare IC_50_ values (determined with MTT assay) between the cold water extract of *I. obliquus* and its protein fraction within the same cell line; between the same extract/fraction across different cell lines (A549 and NL20)

It is important to note that the protein fraction of the *O. sinensis* cold water extract has been reported to exhibit greater cytotoxicity towards NL20 *in vitro* (*30*). In addition, the cold water extract of *L. tigris* was also reported to be cytotoxic to the NL20 cell line. However, intraperitoneal treatment of the protein fraction of cold water extract of *L. tigris* in a mouse xenograft tumour model did not result in any structural damage to the lungs of the mice (*35*). Therefore, the NL20 may be more sensitive to the cytotoxic compounds derived from the fungi. Hence, future studies should include other non-tumorigenic lung cell lines, such as BEAS-2B or HULEC-5a, to confirm the cytotoxic selectiveness of the protein fraction of cold water extract of *I. obliquus*. Moreover, the synergistic interactions between fungal proteins and polysaccharides in a biological system could possibly explain the observed discrepancy between the *in vitro* and *in vivo* results for the cold water extract of fungi. Therefore, future *in vivo* toxicity evaluations of the cold water extract and protein fraction of *I. obliquus* and their effects on the lungs are warranted.

Generally, we found in this study that most of the cold water extracts of fungi are cytotoxic to human cancer cell lines, although there are some exceptions. We hypothesise that the native environment in which the fungi grow may play a crucial role in determining the properties of the bioactive molecules they contain and thus may explain the observed exceptions or inconsistencies in the trends. For example, *I. obliquus* is native to cold circumboreal regions, while *O. sinensis* is found in meadows above 3500 m in the Tibetan region. These fungi thrive at cold or relatively cool temperatures, which means that their proteins, which provide essential homeostasis, are adapted to low temperatures and may not be stable at higher temperatures. This could explain why cold water extraction is more suitable for these fungi to produce more potent cytotoxic compounds against A549 cells. The same theory could also apply to *L. rhinocerus*, which typically grows underground in a shaded area under the canopy. Exposure to higher temperatures may degrade their bioactive compounds. On the other hand, fungi such as *A. camphorata* parasitises endemic camphor trees in Taiwan at altitudes of 400–2000 m above sea level and are more susceptible to temperature fluctuations due to seasonal changes. This could be the reason why the cytotoxic components in *A. camphorata* are preserved at high temperatures. Since our theories are derived from the observations and results, it is necessary to conduct physiochemical characterisation and identification of the chemical composition of the fungal molecules to confirm these claims.

Water is an abundant, cost-effective, non-toxic and environmentally-friendly solvent, and is therefore well-suited for extraction purposes (*36*). Water-based extraction methods have gained interest as environmentally friendly alternatives for efficient extraction of natural compounds, saving time and energy (*37*). Heating water reduces its permittivity, viscosity and surface tension while increasing its diffusivity (*38*, *39*). However, the application of heat during extraction plays a critical role as it can affect the extraction rates and the concentration of compounds, potentially affecting the chemical composition of the final extract (*40*). Interestingly, recent studies have shown that cold water extraction of certain plants and fungi can yield extracts with greater bioactivities than hot water extraction (*12*, *28*, *41*). This suggests that the temperature used during the extraction process can have a significant impact on the stability and effectiveness of the extracted compounds.

Water-based extraction methods are commonly used to extract bioactive compounds from medicinal fungi, such as bioactive polysaccharides and triterpenoids from *Ganoderma lucidum* and bioactive polysaccharide (CP2-S) from *Cordyceps militaris* (*42*, *43*). The extraction of these bioactive compounds from fungi is a critical step in drug development that requires the selection of appropriate and efficient extraction methods (*40*). The product of extraction can be influenced by various factors including but not limited to temperature and duration of extraction, pressure, pH and polarity of the solvent used (*44*). Adjusting the duration of extraction and increasing the temperature are effective modifications that can improve the extraction processes and increase the yield and functionality of the extracted compounds (*45*, *46*). However, the resulting product can also be affected by variables such as prolonged exposure to atmospheric oxygen, oxidative or enzymatic degradation, and intermolecular reactions among mycochemicals (*47*). A notable limitation of our present study, which could serve as a basis for future research, is that the observed results are not only influenced by heat application. Factors such as extraction time and agitation methods can also affect the extraction process and the resulting products. The use of water as the sole extraction solvent may have limited our ability to capture less hydrophilic compounds, potentially overlooking additional bioactive compounds that could be extracted with alternative solvents. In the future, it is essential to explore these variables in more detail. Incorporating additional extraction techniques, such as organic solvent extraction, ultrasonication and supercritical fluid extraction, will broaden our understanding and improve the effectiveness of extraction processes.

### Limitations of study

While this study provides valuable insight into the cytotoxic properties of fungal water extracts, it has some limitations. The stability of these compounds over the 72-hour treatment was not investigated. Without these stability tests, it is uncertain whether the observed cytotoxic effects are due to the extracts as originally prepared or to potentially unstable by-products formed during extended exposure. The focus of this study on a limited number of cancer and non-tumorigenic cell lines also limits the generalisability of the results, as different cell lines may show different responses to the same treatments. In addition, while the *in vitro* results are promising, they may not directly translate to *in vivo* systems, where complex interactions with the immune system, metabolism and bioavailability can alter the observed effects. Finally, the lack of hormonal analysis, particularly in breast cancer cell lines with different hormone receptor status (MCF-7: ER^+^ and PR^+^, MDA-MB-231: ER/PR^-^ and HER2^-^), may limit our understanding of the mechanisms by which the extracts exert their effects. By recognising these issues, future studies are proposed to address these limitations by incorporating a broader range of extraction solvents, stability testing, more diverse cell lines, *in vivo* testing and detailed hormonal impact assessments to fully explore the therapeutic potential of these fungal extracts.

## CONCLUSIONS

In conclusion, our study shows that the cold water extracts generally exhibit increased cytotoxicity towards certain human cancer cell lines in comparison with the heat-assisted water extracts. It is worth noting that bioactivity may vary due to the isolation of different compounds with different properties, emphasising that each extraction method has its own advantages. This study has highlighted the potential of the cold water extract as a good source of proteins and phenolic compounds, while the heat-assisted extraction can be used to obtain carbohydrates. Our study also emphasises the importance of extraction conditions, especially heating, in influencing the bioactivity of fungal extracts and the complexity of extracting their bioactive compounds to utilise their full therapeutic potential. Therefore, additional studies are required to validate our findings and investigate various factors and conditions that may affect the bioactivities of these fungal extracts. Further research will contribute to a deeper understanding of the optimal extraction methods and conditions required to isolate specific bioactive compounds for future drug development from natural products.

## Appendix

**Table S1:** Half maximal inhibitory concentration (IC_50_) of fungal extracts against A549 lung carcinoma, and MCF-7 and MDA-MB-231 breast carcinoma cell lines after 72 h of treatment in MTT assay

		IC_50_/(µg/mL)		
Fungus	Extraction	A549	MCF-7	MDA-MB-231
*L. rhinocerus*	CWE	(848±71)^a^	(352±10)^a^	(703±20)^a^
	HAWE_supernatant_	>2000	(1838±122)^b^	>2000
	HAWE_precipitate_	>2000	>2000	>2000
*O. sinensis*	CWE	(774±81)^a^	>2000	>2000
	HAWE_supernatant_	>2000	(1269±224)^c^	>2000
	HAWE_precipitate_	>2000	>2000	>2000
*A. camphorata*	CWE	(1527±83)^b^	>2000	>2000
	HAWE	(1468±166)^b^	>2000	>2000
*I. obliquus*	CWE	(701±28)^a^	>2000	(1401±120)^b^
	HAWE	>2000	>2000	>2000
*P. linteus*	CWE	(1980±21)^c^	(1110±48)^c^	>2000
	HAWE	>2000	>2000	>2000
*M. purpureus*	CWE	>2000	>2000	>2000
	HAWE	>2000	>2000	>2000

Data are presented as mean value±S.D. (*N*=9). Different letters in superscript in the same column denote significant differences (p˂0.05). Comparison of IC_50_ values between the fungal extracts within the same cell line was done using one-way ANOVA followed by Tukey’s HSD *post-hoc* test (for A549 and MCF-7 cell lines) and Student’s *t*-test (for MDA-MB-23 cell line). CWE=cold water extraction, HAWE=heat-assisted water extraction
